# The impact of two-year community-wide pharmacy interventions on the public’s knowledge, attitudes, beliefs and behaviors about pharmacists as immunizers

**DOI:** 10.1186/s12889-025-24906-3

**Published:** 2025-12-05

**Authors:** Donna M. Halperin, Antonia M. Di Castri, Jennifer E. Isenor, Wilfrid Kouokam Lowe, Lingyun Ye, Melissa S. Kervin, Carrie A. Whittle, Scott A. Halperin

**Affiliations:** 1 Canadian Center for Vaccinology, Dalhousie University, IWK Health, Nova Scotia Health, Halifax, NS Canada; 2 Rankin School of Nursing, St. Francis Xavier University, Antigonish, NS Canada; 3 College of Pharmacy, Dalhousie University, Halifax, NS Canada; 4 Department of Pediatrics, Dalhousie University, Halifax, NS Canada; 5 Department of Microbiology and Immunology, Dalhousie University, Halifax, NS Canada

**Keywords:** Pharmacist, Interventions, Immunizers, Public, Vaccines, Uptake, Behaviors, Knowledge

## Abstract

**Background:**

Immunization rates for vaccine-preventable diseases remain well below targets worldwide, leading to unnecessary morbidity and mortality, and heavy economic burdens to healthcare systems and workforces. One promising avenue to improve vaccine coverage is through the public’s acceptance and use of pharmacists as immunizers; yet, at present, pharmacists remain underutilized for vaccine uptake by the public. Further study is needed on how to improve the perceptions and use of pharmacists as immunizers.

**Methods:**

Between 2017 and 2019, pharmacy interventions were conducted in communities in Nova Scotia and New Brunswick, Canada, with 23 intervention pharmacies and 21 non-intervention pharmacies. Interventions focused on seven vaccines, and included activities such as pharmacists providing counsel to patients (registered patients and walk-ins), online messaging, and pharmacy posters. Following the interventions, online surveys were conducted among adult residents in the communities (*n* = 992) to assess the public's knowledge, attitudes, beliefs, and behaviors (KABB) regarding pharmacists as immunizers. Data were analyzed using Fisher’s exact tests, multiple regressions, and Spearman’s ranked correlations.

**Results:**

As compared to non-intervention communities, respondents in intervention communities had improved awareness of their vaccination status and increased self-reported uptake for meningococcal ACWY/C and travel vaccines, and reduced uptake of the shingles vaccine (*P* < 0.05). From combined analysis of non-intervention and intervention communities, willingness to receive a vaccine was strongly associated with being offered that vaccine in the past (*R* = 1.0, *P* < 10⁻⁷) and with awareness of recommendations from the National Advisory Committee on Immunization (NACI) (*R* = 0.79, *P* < 10⁻⁷). Less than 13% of respondents were aware that pharmacists provide the pertussis vaccine, while 90.7% reported they would receive all studied vaccines if free of charge. Respondents without access to a family physician had three-times higher odds of using the internet for vaccine information compared to those with a physician.

**Conclusions:**

The KABB of respondents in intervention and non-intervention communities provided insights into improving the public’s use of pharmacists as immunizers. Future strategies may include pharmacists proactively offering vaccines and counsel on Public Health vaccine guidelines to patients, increasing the public’s awareness about vaccines provided by pharmacists (through healthcare providers and social media), and expanding public funding for vaccines with low uptake.

**Supplementary Information:**

The online version contains supplementary material available at 10.1186/s12889-025-24906-3.

## Background

Vaccine-preventable infections remain a global health concern, and immunization rates are well below target levels for numerous diseases, including in Canada [1–4]. In addition to reducing morbidity and mortality for common diseases affecting individuals, vaccination has broad community health and economic benefits such as reduced burden on the healthcare system, enhanced worker productivity and lower worker absenteeism, including for healthcare workers (e.g., for influenza) [1, 5, 6]. To meet public health targets, expanding vaccine coverage in communities has been a primary goal of health agencies [4, 7]. One underutilized avenue to enhancing vaccine coverage among the general public may be through the expansion of vaccine providers, particularly the use of pharmacists as immunizers [1–3].

Community pharmacists may overcome common barriers to vaccination as they are available for a wider range of hours than is typically the case for family physicians or nurses, are accessible through community-based retail locations, and are often the most convenient option for individuals from diverse demographic, cultural, and economic backgrounds [1, 8]. Further, for individuals without a primary care physician, a pharmacist may provide the only accessible avenue to obtaining vaccinations [3, 9, 10]. In Atlantic Canada (including Nova Scotia (NS) and New Brunswick (NB)) for example, due to sustained physician shortages, about 12–17% of residents have no access to a family physician or nurse practitioner [11], that disproportionately impacts healthcare access for younger people (18–24 years) and those with the lowest incomes [12]. Given their wide accessibility, pharmacists are well positioned to reach a diverse range of individuals within a community that would otherwise remain unvaccinated against vaccine-preventable diseases [1, 9, 10, 13].

Pharmacists’ roles in providing vaccinations have expanded over the last two decades worldwide (e.g., UK, Ireland, Canada, USA, Portugal) [3, 14–16]. Within Canada, pharmacists in all ten provinces and one of three territories (Northwest Territories and Nunavut territories are exceptions) have been trained and authorized to administer numerous vaccines for adults, and in some regions, for children [15]. Like in other countries, in Canada, the inclusion of pharmacists as vaccine providers has been associated with an increase in community or national immunization rates, with estimates of a 2.2% increase to more than an 18% increase [8, 17–21], but nonetheless pharmacists remain underutilized as vaccinators [1–4]. In fact, vaccine coverage remains well below public health targets in Canada; for example, for influenza, the coverage goal in adults 18–64 years of age is 80% of the population. However, only 43% of the public were vaccinated in Canada during 2022–2023, which is well below the national target [4, 7]. This gap highlights the importance of exploring accessible, community-based, strategies to enhance vaccine uptake such as utilizing pharmacists as immunizers. In addition to influenza, pharmacists may provide other vaccines recommended by the National Advisory Committee on Immunization (NACI) such as those for pertussis (Tdap), meningococcus (meningococcal ACWY/C and B), shingles (herpes zoster), and travel (e.g., hepatitis A, hepatitis B, and/or typhoid vaccines) [22, 23], providing a promising and underrealized opportunity to improve coverage for efficacious vaccines. Currently, more data are needed to understand how to improve the public’s use of pharmacists for their vaccinations.

One research approach to assess whether pharmacists may influence vaccination acceptance and/or uptake is through pharmacy interventions, whereby pharmacists take measures to reach out to patients and provide counsel about or promote vaccination [3, 24]. For example, a pharmacy intervention study in which pharmacists provided educational information to older adults about the influenza vaccine showed a 1.25 fold increase in vaccine uptake as compared to control groups without an intervention [25]. Similar findings have been reported in other influenza intervention studies, which demonstrated improvements in vaccine coverage through pharmacist-led education, reminders, or on-site immunization campaigns [26, 27]. An intervention whereby pharmacists provided hospital inpatients written and verbal vaccine information on influenza and/or pneumococcal vaccines found improved uptake of both types of vaccines among those patients [28]. Similarly, increased vaccination rates were obtained for a pharmacist intervention among people with cancer ≥ 65 years of age, where pharmacists met with patients and made vaccination recommendations; receipt of vaccinations was improved by >27% for pneumonia and influenza in the intervention group, and 0% of controls [29] (for reviews of pharmacy interventions, see [3, 24]). These types of targeted pharmacy intervention studies, however, have often focused on known pharmacy or hospital patients and examined only one or two vaccines, typically measuring vaccine uptake in those same individuals or a parallel control group [3]. This narrow focus does not address whether a broader, community-wide approach, to interventions across multiple pharmacies may shape the public’s uptake of a wide range of vaccines. Further, there remains a limited understanding about the public’s knowledge, attitudes, beliefs, and behaviors (KABB) about vaccination by pharmacists in intervention and non-intervention communities. Community-based research of KABB may provide valuable insights into how to improve vaccine uptake, and in turn, vaccine coverage of the general public.

The aim of the present study is to enhance our understanding of the KABB pertaining to pharmacists as immunizers among adult residents of communities following two-years of vaccine-related interventions, either at an intervention or non-intervention site, as a means to identify strategies to improve the public’s use of pharmacists for immunization. The study was conducted in four communities located in two eastern provinces of Canada, NS and NB.

## Methods

### Study setting and interventions

The non-intervention sites included the Canadian cities/towns of Kentville, NS and Moncton, NB and the intervention sites included Antigonish, NS and Saint John, NB, whereby each city/town included their respective surrounding townships. Thus, as shown in Table 1, one location per province was an intervention site and one was a non-intervention site. At the time of the study each community had at least 30,000 adult residents, representing a substantial population size for a city/town in each of these two provinces, and was deemed to have lack of spillover in pharmacy services with other communities [30]. Each site is separate geographically and has their own set of health centers [30, 31].

**Table 1:** The number of survey respondents per study site for non-intervention and intervention communities

Survey period	Non-intervention communities			Intervention communities		
	(*n* = 21 pharmacies)			(*n* = 23 pharmacies)		
	Kentville NS	Moncton NB	Total	Antigonish NS	Saint John NB	Total
2020-2021 (Post-intervention)	377	135	512	341	139	480

Each city/town includes the surrounding townships; Number of respondents was *n* = 992

Recruitment of pharmacies is described in [30] and the final n of pharmacies are shown in the table

A total of 21 non-intervention pharmacies (7 in NS, 14 in NB) and 23 intervention pharmacies (11 in NS, 12 in NB) participated in our study between April 3, 2017 (most started in September 2017) and November 30, 2019 (Table 1), comprising approximately two years of pharmacy interventions. Details on the recruitment of pharmacies are described in our prior methods report [30]. Pharmacists in the intervention communities implemented outreach strategies related to seven vaccine types that were studied in the follow-up surveys:

1. Seasonal influenza [23];
2. High-dose influenza (for adults aged 65+ [23, 32]);
3. Meningococcal ACWY/C;
4. Meningococcal B (for individuals under 25 years of age [23]);
5. Tdap (tetanus, diphtheria, and pertussis—recommended once in adulthood and during each pregnancy [23]);
6. Shingles (herpes zoster, for adults aged 50+ [23, 33]), and;
7. Travel vaccines (hepatitis A, hepatitis B, and typhoid).

The pharmacy interventions involved active outreach by pharmacists to patients (registered patients and walk-ins) within the pharmacy setting including combining vaccine promotion with other services (e.g., back-to-school events, allergy recommendations, smoking cessation, prescription refills), making targeted vaccine recommendations, participating in immunization weeks, and using social media and signage provided by the study team [30]. Interventions were directed towards all visitors to the pharmacy including registered patients, walk-ins, people seeking over the counter medications or pharmacy advice, and those exposed to in-pharmacy or online intervention materials. Intervention pharmacists received training in-person or by video conference; further details on the study and the enhanced outreach strategies have been described previously [30]. Pharmacists were permitted to use their professional discretion with respect to which interventions were most suited to their pharmacy and patients and could delegate some activities to trained staff including pharmacy technicians and assistants, e.g., applying posters and reminders for influenza vaccines. Pharmacies in non-intervention communities continued their standard immunization practices without any study-related training or outreach.

### Population sample

The surveys were conducted between October 23, 2020 and July 5, 2021 in the non-intervention and intervention communities; recruitment of participants was ongoing during this period. All individuals residing in a study site and that were ≥ 18 years of age were eligible to respond to the survey (for each of the four study sites). Individuals were invited through social media, local print and online ads, posters at pharmacies and two rounds of emails to university list-servers. The respondents could enter a prize draw for one $100 gift card [31]. Advertisements provided the web address for the online surveys [31]. To access the survey, each respondent had to electronically confirm that they read the online consent form and agreed to voluntarily participate in the survey (options available were to agree or decline). All members of the community could respond to the survey, including those who visited pharmacies or did not visit. Although individual-level exposure was not required or verified, the interventions were implemented across all participating pharmacies in the intervention communities (Table 1), and most residents who visited pharmacies were likely exposed. Frequency of pharmacy visits of the respondents was collected in the surveys to help interpret variation in public exposure.

### Survey Instrument

A previously used and validated online survey was administered to adult members of the public residing in each community [31]. The survey was designed using a formative process informed by the Theory of Planned Behavior with target constructs from the Health Belief Model [34–37], and according to the principles of survey design [38]. Where relevant to the current study objectives, we incorporated questions from previously developed surveys by our investigator team. These survey items had been pre-screened by healthcare professionals and scientists, piloted in earlier studies, and used successfully in prior published research (e.g., [39–41]). The questionnaire was evaluated by a panel of six experts comprised of nurses, pharmacists, and infectious disease physicians at the Canadian Center for Vaccinology (CCfV), and we retained those questions with high relevancy and validity scores, and those with high consistency/reliability in participant responses when repeated over time [31].

The final questionnaire contained 98 items, including yes/no/I don’t know, Likert-scale items assessing strength of agreement and multiple-choice questions. The survey included queries on awareness of vaccine availability, demographics, attitudes about specific vaccines, social pressures to vaccinate, access to vaccines, intention to vaccinate, and the comfort and prior use of pharmacists for vaccination. There were also queries about a range of vaccines including influenza, meningococcal ACWY/C and B, whooping cough/pertussis (Tdap), and high-dose influenza [31]. Additions to the prior validated survey in the present study [31] included questions about travel vaccines (pooled for hepatitis A, hepatitis B, and/or typhoid vaccines) and the shingles vaccine (herpes zoster) for those ≥ 50 years of age, and three queries on COVID-19 vaccines. As the survey period occurred during the COVID-19 pandemic, respondents were asked to focus their thoughts and opinions on the period before March 2020 (the start of the pandemic). However, given the timing of data collection, we cannot exclude the possibility that respondents’ responses were influenced by their experiences during the COVID-19 pandemic. For methods reporting we used guidelines from the Consensus-Based Checklist for Reporting of Survey Studies (CROSS) [42].

The population sample and surveys were designed to understand: (1) differences in KABB between communities that experienced pharmacy interventions (intervention sites) and those that did not (non-intervention sites), and; (2) KABB patterns that were consistent across both types of communities, which may further insights for improving pharmacist-led immunization uptake.

### Statistical analysis

For statistical analysis, the two non-intervention and two intervention communities were each defined as non-intervention and intervention groups (Table 1). Survey questions provided either categorical responses (binary (e.g., yes/no) and nominal responses) or ordinal (discrete) responses on the Likert scale. The percentage for each reply class per query was determined along with the 95% confidence interval (CI) using the Clopper Pearson method. Fisher’s exact tests were performed to compare responses between the non-intervention versus intervention groups. A sample size of 400 individuals provides a 95% confidence interval (CI) of a maximum ± 5% of the value (percent) obtained for any survey question.

For selected questions deemed suitable for follow-up analysis, based on scientific literature and the past research by the study team [31, 39–41], and their relevance to KABB, we used regression models to examine whether responses could be predicted by other survey items. Logistic regression was performed to predict binary and non-ordered responses, and ordinal logistic regression was used for ordered responses. Multiple regression was used to ascertain whether the dependent variable was dependent on two or more independent variables, which are shown in the outcome tables. These included, for example, whether the sources of vaccine information (from a family physician, pharmacist, nurse, media, or the internet) could be predicted by factors such as age, frequency of pharmacy visits, or access to a family physician. Outcome (dependent) variables were predicted using multivariable regression wherein demographic, knowledge, attitudinal, and belief-based variables were used in a stepwise backwards elimination model (as described in [43]). Odds ratios were determined to reveal the directional relationship between the dependent and independent variable (for those variables with *P* < 0.05). Spearman’s ranked correlation coefficients (non-parametric) were used to assess correlations. The analysis Software was SAS^®^ version 9.4 [43]. P-values of < 0.05 were considered statistically significant for all analyses.

## Results

A total of 992 adults were surveyed: 512 respondents resided within the non-intervention communities and 480 resided within the intervention communities (Table 1). With respect to demographics, among the 992 survey respondents, 76.1% were female (Table 2). All age groups were represented; 70.7% of respondents were under age 50, with 42% younger than 25 years (Table 2). In turn, 22.8% of respondents were 55 years of age or older. The highest level of education obtained was high school for 33.5% of respondents, while a majority (65.6%) had obtained post-high school education (46.1% had a Bachelor’s, Master’s, or a Doctoral degree). Annual household incomes varied substantially; 19.1% of respondents fell within the lowest income category of under $39,000, while 21.1% were in the highest (≥$125,000), and the remainder had intermediate incomes or preferred not to answer (59.8%). No demographic differences were observed between the intervention and non-intervention groups.

**Table 2:** Demographic characteristics of the survey respondents

Demographic Characteristic	*n*	Percent	95% CI
**Gender**			
Male	228	23	19.9–26.3
Female	755	76.1	72.7–79.2
Other	9	0.9	0.4–2.0
**Age**			
18 to 24 years of age	417	42	37.9–46.3
25 to 34 years of age	121	12.2	9.7–15.3
35 to 44 years of age	101	10.2	7.9–13.1
45 to 49 years of age	62	6.3	4.5–8.7
50 to 54 years of age	65	6.6	4.7–9.0
55 to 64 years of age	133	13.4	10.8–16.6
65 years of age or older	93	9.4	7.2–12.2
**Highest completed level of education**			
Elementary	2	0.2	0.0–1.1
High School	332	33.5	29.5–37.7
College or pre-university	126	12.7	10.1–15.9
University certificate or diploma	67	6.8	4.9–9.3
Bachelor’s degree	242	24.2	20.9–28.3
Master’s degree	126	12.7	10.1–15.9
Doctorate degree	91	9.2	7.0–12.0
I prefer not to answer	6	0.6	0.2–1.7
**Annual household income**			
Under $39,000	189	19.1	16.0–22.6
$39,000 to $70,000	169	17.0	14.1–20.4
$70,000 to $90,000	126	12.7	10.2–15.8
$90,000 to $125,000	153	15.4	12.6–18.7
$125,000 or more	209	21.1	17.9–24.7
I prefer not to answer	146	14.7	12.0–17.9
**Place of residence**			
Nova Scotia	718	72.4	69.1–75.4
New Brunswick	274	27.6	24.6–30.9

### Differences between non-intervention and intervention responses: vaccine behaviors

Survey responses showed that the self-reported receipt of vaccines varied extensively among vaccine types for respondents in both the non-intervention and intervention groups. Specifically, the annual influenza vaccine was the most commonly received by respondents, with a percentage of at least 62.1% (in both groups) and the least commonly received was meningococcal ACWY/C (≤ 28.6, Table 3). For each query about the receipt of a particular vaccine, we assessed whether participant responses (possible responses were “yes”, “no”, or “I don’t know”) were associated with the non-intervention and intervention status. We found that the participant responses were statistically significantly associated with the intervention/non-intervention status for three vaccines, namely travel (Fisher’s test *P* = 0.024), meningococcal ACWY/C (*P* = 0.006), and the shingles vaccines (*P* = 0.045, Table 3).

**Table 3:** Vaccination behavior for respondents from the non-intervention and intervention communities

Vaccination behavior	Response	Non-intervention			Intervention			*P*-value	PP diff	Percent diff
		N	Percent	95% CI	N	Percent	95% CI			
Received the influenza vaccine^a^	Yes	306	62.1	(57.1–66.8)	304	66.8	(61.7–71.6)	0.136	4.7	7.57
	No	187	37.9	(33.2–42.9)	151	33.2	(28.4–38.3)		−4.7	−12.40
Ever received meningococcal (meningitis) ACWY/C vaccine	Yes	118	24.0	(19.7–28.9)	130	28.6	(23.8–33.9)	0.006	4.6	19.17
	No	138	28.0	(23.5–33.1)	154	33.8	(28.8–39.3)		5.8	20.71
	I don’t know	236	48.0	(42.6–53.4)	171	37.6	(32.3–43.1)		−10.4	−21.67
Ever received meningococcal (meningitis) B vaccine	Yes	62	28.3	(21.6–36.1)	61	36.7	(28.4–46.0)	0.178	8.4	29.68
	No	35	16.0	(10.9–22.8)	27	16.3	(10.6–24.2)		0.3	1.88
	I don’t know	122	55.7	(47.6–63.5)	78	47.0	(38–56.2.2)		−8.7	−15.61
Ever received Tdap vaccine	Yes	201	40.9	(35.7–46.2)	210	46.3	(40.7–51.9)	0.149	5.4	13.21
	No	187	38.0	(32.9–43.4)	167	36.8	(31.6–42.3)		−1.2	−3.16
	I don’t know	104	21.1	(17.1–25.9)	77	17.0	(13.2–21.6)		−4.1	−19.43
Ever received travel vaccine^c^	Yes	262	53.3	(47.9–58.6)	268	59	(53.4–64.4)	0.024	5.7	10.69
	No	171	34.8	(29.8–40.0)	154	33.9	(28.8–39.4)		−0.9	−2.59
	I don’t know	59	12.0	(8.9–15.9)	32	7.0	(4.7–10.5)		−5	−41.67
Ever received herpes zoster (shingles) vaccine^d^	Yes	49	36.8	(27.7–47.0)	51	34.2	(25.8–43.8)	0.045	−2.6	−7.07
	No	75	56.4	(46.2–66.0)	96	64.4	(54.8–73)		8	14.18
	I don’t know	9	6.8	(3.2–13.8)	2	1.3	(0.3–5.9)		−5.5	−80.88

Fisher’s exact test* P<0.05 *are provided

The 95% confidence intervals (CI) are show

^a^During the past fall or winter. Note that influenza only had “yes” or “no” possible responses, High-dose influenza vaccine was excluded in table due to low n (≤ 44)

^b^ Question for meningitis B was for those 18–24 years of age

^c^ hepatitis A, hepatitis B, and/or typhoid vaccines

^d^ Question was only for those 50 years of age and above

We further considered how responses about self-reported vaccine receipt varied for the three vaccines with statistically significant differences between the non-intervention and intervention groups (Table 3). Survey queries asked whether respondents had received each vaccine at any point in the past; responses reflect self-reported vaccine uptake, not time of administration. For the travel vaccines, the largest percentage point (PP) difference was observed for the “yes” response, which was 5.7 PP higher for the intervention (percent uptake was 59.0%) than non-intervention group (53.3%; Table 3; see tables for the 95% confidence intervals herein). This increase in “yes” was associated with a decline in the “I don’t know” response (of 5.0 PP, Table 3), and thus the improvement in travel vaccines uptake in the intervention group may indicate greater receipt of the vaccines, combined with an improved awareness of having received travel vaccines in the past. For the meningococcal ACWY/C vaccine, the largest difference was observed for the “I don’t know” response, that had a decline of 10.4 PP in the intervention as compared to the non-intervention group and had a roughly similar increase in the “yes” (4.6 PP) and “no” (5.8 PP) responses (in the intervention group). The higher percentage of vaccine receipt observed in the intervention than the non-intervention group for meningococcal ACWY/C (uptake was 28.6% versus 24.0% respectively) may indicate an overall improvement in vaccine coverage combined with improved awareness of having received this vaccine. Finally, the shingles vaccine, which was an age-restricted query (≥ 50 years of age), had the largest increase in PP (of 8.0) observed for the “no” response in the intervention group (note that the “I don’t know” response was not considered here as *n* ≤ 9 for both groups, Table 3), implying the intervention was linked to lower uptake of this vaccine. However, the “no” response was received from a majority of respondents for both the intervention and non-intervention groups for the shingles vaccine at 64.4% and 56.4% respectively, indicating hesitancy was common to both groups.

### Analyses of combined non-intervention and intervention surveys

While the intervention status was associated with vaccine uptake, many KABB queries were similar between non-intervention and intervention groups, and combined analysis of responses from both groups yielded the findings described below.

### Vaccine awareness: being offered a vaccine is linked to high uptake

**Fig. 1:**
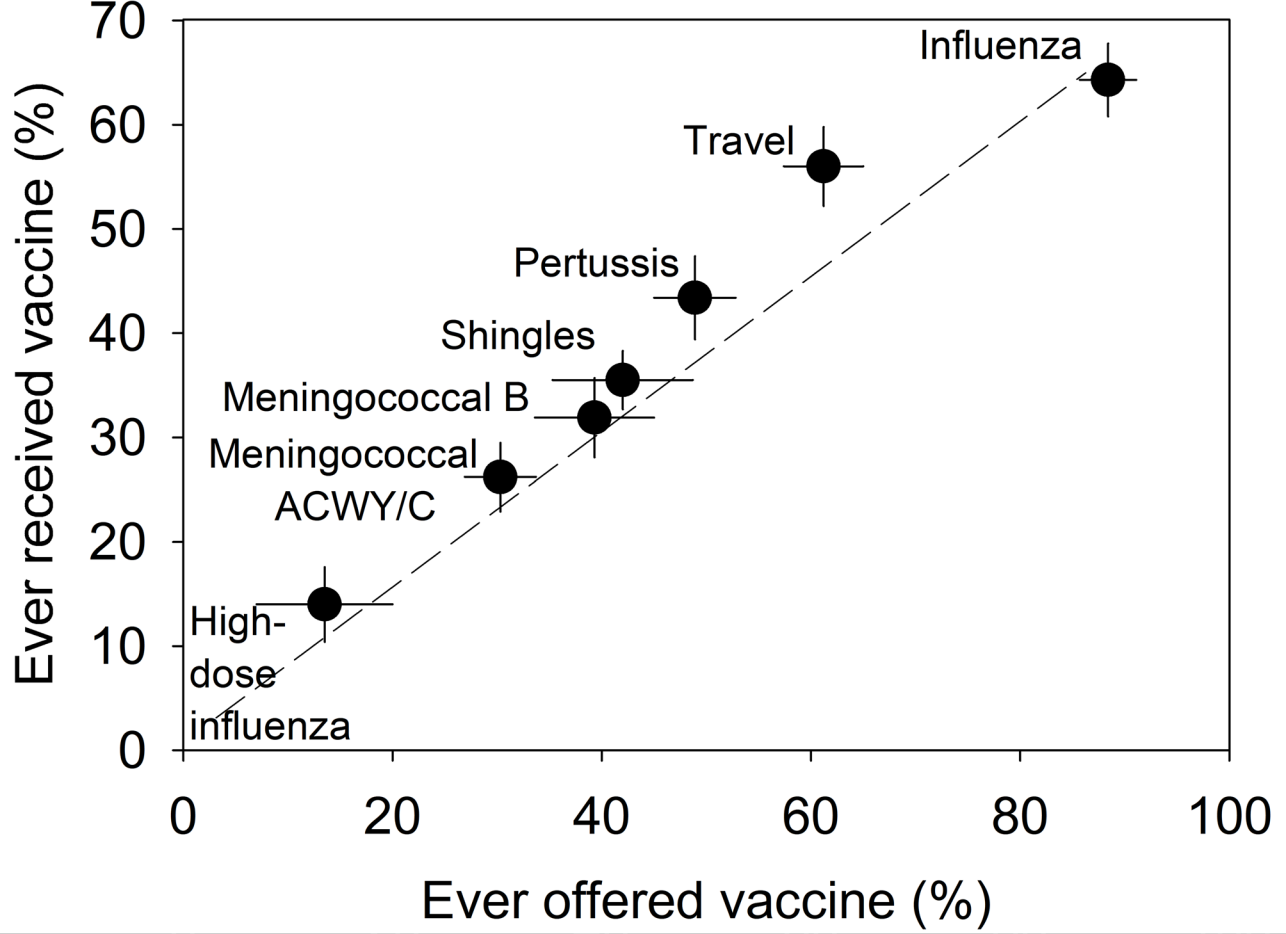
Percent of respondents that received the vaccine versus previously offered the vaccine. Bars = 95% confidence intervals. Spearman’s *R* = 1.0, *P* < 10^−7^

To assess the association between vaccine uptake and a history of being offered the same vaccine, and while recognizing some differences in receipt of vaccines (Table 3), we pooled the results for the non-intervention and intervention respondents to study common patterns shared among both groups. As shown in Fig. 1, we observed a stepwise association between the percent of respondents that had been offered the vaccine in the past and the percent that had received a vaccine (i.e., those vaccines more commonly offered within the communities tended to also have greater uptake; 95% confidence intervals shown in figure). For example, the influenza vaccine had the highest percentage of respondents offered the vaccine at 88.4% and the highest uptake at 64.3% (Fig. 1). Following a stepwise decline, a similar relationship was observed between being offered a vaccine and uptake for the travel vaccine (offered = 61.2%, received = 56.0%), pertussis (48.9% and 43.4% respectively), shingles (42% and 35.5%), meningococcal B (39.2% and 31.9%), meningococcal ACWY/C (30.3% and 26.2%), and for high-dose influenza (14.0% and 14.0%) (Spearman’s *R* = 1.0, *P* < 10^−7^). Moreover, using multivariate analysis (and odds ratios), we found that receipt of a vaccine among study respondents was associated with increased odds of being offered a vaccine in the past (see for examples, influenza, meningococcal ACWY/C, and Tdap, *P* < 0.05, Table S1). We found that 92.9%, 88.9%, and 83.2% of all study respondents agreed that they would receive a vaccine if it was offered by a physician, nurse, or pharmacist, respectively, further supporting the above data that being proactively offered a vaccine by a health professional is associated with high acceptance rates and willingness to receive a vaccine.

### Awareness of NACI recommendations is associated with intention to receive a vaccine

**Fig. 2:**
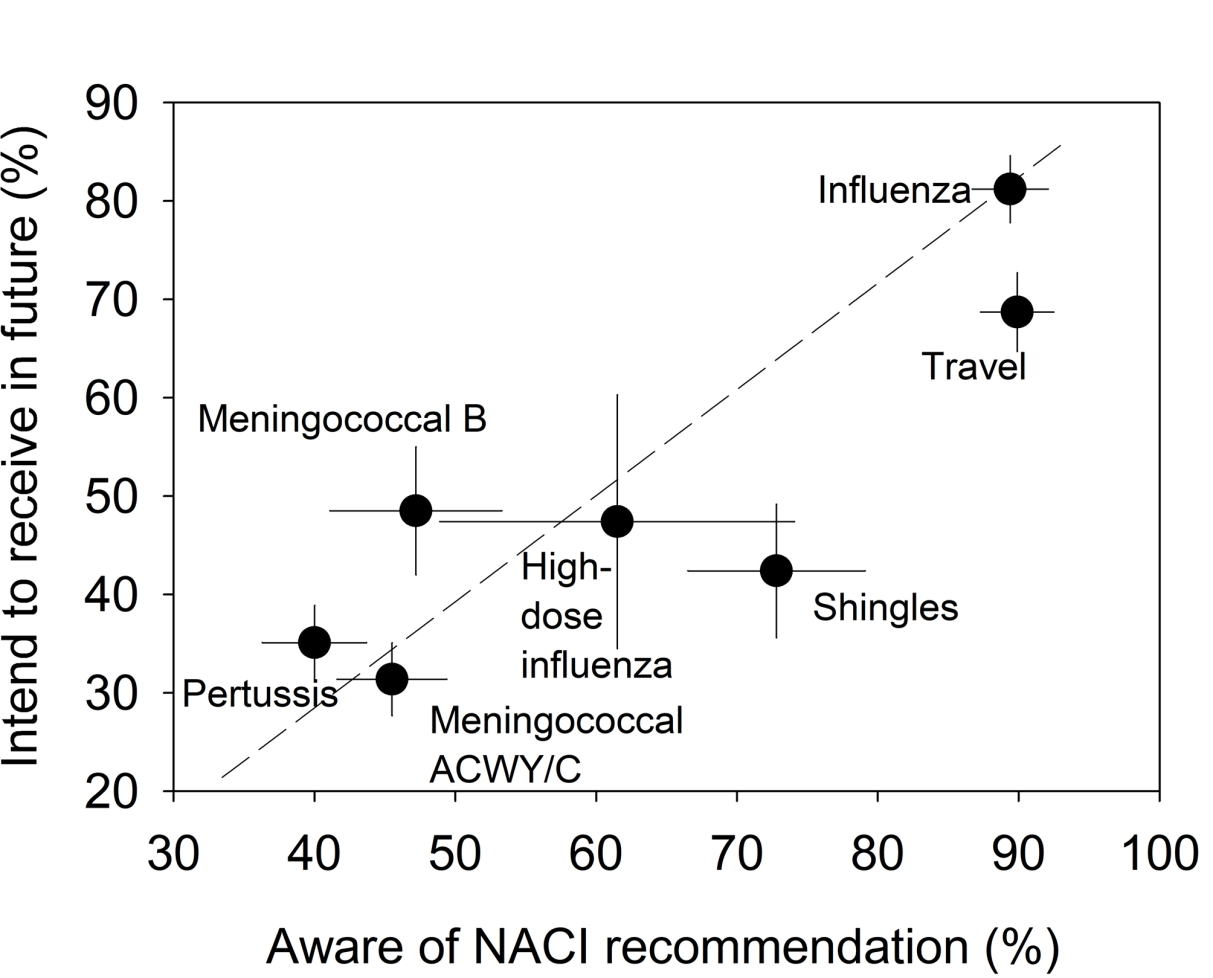
Percent of respondents that intend to receive vaccine versus those aware of NACI recommendation. NACI = National Advisory Committee on Immunization (public health). Bars = 95% confidence intervals. Spearman’s *R* = 0.79, *P* < 10^−7^

Respondents’ awareness (or, knowledge in KABB) of the NACI (public health) recommendations for a vaccine was associated with the intention to receive the vaccine in the future (Fig. 2, Spearman’s *R* = 0.79, *P* < 10^−7^; and each may be innately tied to being offered a vaccine and uptake (see below, Table S1)). The percent of respondents aware of the NACI recommendations (89.4%) and the percent intending to receive a vaccine (81.2%) were each highest for the influenza vaccine. The travel vaccine, high-dose influenza, meningococcal B and ACWY/C, and Tdap all showed a similar association between the percent of respondents aware of the NACI recommendations and the percent interested in receiving the vaccine (Fig. 2). One exception to this pattern was the shingles vaccine, where 72.8% of respondents (query was restricted to those 50 years of age and older) expressed awareness of the NACI recommendations, but only 42.4% planned to receive the vaccine (Fig. 2). The disparity is indicative that there is a hesitancy for receiving the shingles vaccine, even though most studied individuals were aware of the NACI recommendation (Fig. 2) and believed that shingles is a threat to their health (observed for 82.8% of respondents, Table S2). Nonetheless, as shown in Fig. 1, pro-actively offering the shingles vaccine by health professionals was linked to improved uptake. It is worth noting that the history of being offered a vaccine (Fig. 1) may be innately interrelated to an awareness of NACI recommendations (Fig. 2; see for example, Tdap where both factors are linked to vaccine uptake in Table S1) among respondents if for example, both resulted from interactions with healthcare providers, and thus each may contribute towards respondents’ prior receipt or intent to receive the vaccines (Figs. 1 and 2, Table S1).

### Awareness of vaccines administered by pharmacists

Table 4 provides a summary of respondents’ KABB regarding pharmacists as immunizers, and the results were similar between the non-intervention and intervention groups. Respondents from the non-intervention and intervention groups each had high awareness that pharmacists are trained to administer vaccines (92.9% and 92.3% respectively), agreed that pharmacists have enough training to provide vaccines (88.8% and 85.2%, respectively), and were comfortable with pharmacists as immunizers (86.6% and 85.6% respectively, P = not statistically significant (NSS) for each contrast of non-intervention and intervention). Moreover, for the non-intervention and intervention groups, 83.7% and 82.6% respectively visited a pharmacy at least twice year while 80.6% and 79.8%, agreed that vaccination by a pharmacist was convenient (Table 4; P = NSS per contrast). A majority of respondents reported having received a vaccination from a pharmacist in the past, 61.3% in non-intervention communities and 59.6% in intervention communities (P = NSS).

**Table 4:** Knowledge, attitudes, beliefs and behaviors about pharmacists as immunizers for the non-intervention and intervention groups

	Non-intervention			Intervention			*P*-value
	*N*	Percent	95% CI	*N*	Percent	95% CI	
**Frequency of visits to pharmacy**							
Less than once a year	78	16.3	(12.5–21.0)	77	17.4	(13.3–22.3)	0.464
2–6 times a year	231	48.3	(42.7–54.0)	229	51.7	(45.8–57.6)	
7–12 times a year	140	29.3	(24.4–34.7)	109	24.6	(19.9–30.0)	
More than once a month	29	6.1	(3.9–9.4)	28	6.3	(4.0–9.9.0.9)	
**Aware that pharmacists are trained to administer vaccines**							
Yes	444	92.9	(89.8–95.1)	408	92.3	(89–94.7.7)	0.801
No	34	7.1	(4.9–10.2)	34	7.7	(5.3–11)	
**Aware of pharmacists’ authority to administer the following vaccin**e ^a^							
Influenza	350	68.4	(64.1–72.4)	330	68.8	(64.4–72.9)	0.945
Pertussis	65	12.7	(9.9–15.9)	57	11.9	(9.1–15.1)	0.7
Meningococcal (meningitis) ACWY/C	49	9.6	(7.2–12.5)	40	8.3	(6.0–11.2.0.2)	0.507
Meningococcal (meningitis) B	47	9.2	(6.8–12)	39	8.1	(5.8–10.9)	0.574
Travel vaccines (hepatitis A, hepatitis B, and typhoid vaccines)	170	33.2	(29.1–37.5)	157	32.7	(28.5–37.1)	0.893
Herpes zoster (shingles)	121	23.6	(20–27.6.6)	128	26.7	(22.8–30.9)	0.273
**Ever received a vaccine by a pharmacist**							
Yes	293	61.3	(55.9–66.5)	264	59.6	(53.9–65)	0.706
No	175	36.6	(31.5–42)	172	38.8	(33.5–44.5)	
I don’t know	10	2.1	(1.0–4.3.0.3)	7	1.6	(0.7–3.7)	
**Convenient for me to receive my vaccines from my pharmacist**							
Disagree	13	2.8	(1.5–5.3)	21	4.9	(2.9–8.0.9.0)	0.252
Neither agree nor disagree	77	16.6	(12.9–21.2)	66	15.3	(11.6–19.9)	
Agree	373	80.6	(75.8–84.6)	344	79.8	(74.8–84.0)	
**Comfortable receiving my vaccines from a pharmacist**							
Disagree	26	5.6	(3.5–8.7)	32	7.4	(4.9–11.0)	0.509
Neither agree nor disagree	36	7.8	(5.3–11.3)	30	6.9	(4.5–10.5)	
Agree	402	86.6	(82.4–90.0)	370	85.6	(81.1–89.2)	
**Pharmacists have enough training to give vaccines**							
Disagree	14	3.0	(1.6–5.6)	20	4.6	(2.7–7.7)	0.242
Neither agree nor disagree	38	8.2	(5.6–11.8)	44	10.2	(7.2–14.2)	
Agree	412	88.8	(84.8–91.8)	368	85.2	(80.6–88.8)	
**I would feel more comfortable receiving my vaccines in a pharmacy if my physician recommended it**							
Disagree	82	17.7	(13.8–22.3)	95	22.0	(17.6–27.1)	0.204
Neither agree nor disagree	198	42.7	(37.3–48.2)	184	42.6	(37–48.3.3)	
Agree	184	39.7	(34.4–45.2)	153	35.4	(30.1–41.1)	
**I would feel more comfortable receiving my vaccines in a pharmacy if a public health nurse recommended it**							
Disagree	87	18.8	(14.8–23.5)	101	23.4	(18.9–28.6)	0.23
Neither agree nor disagree	224	48.3	(42.8–53.8)	194	44.9	(39.3–50.7)	
Agree	153	33.0	(28–38.4.4)	137	31.7	(26.6–37.3)	
**Have any of your healthcare providers (e.g. family physician, nurse, pharmacist) informed you of vaccines you should receive?**							
Yes	261	54.6	(49.1–60.0)	250	56.4	(50.7–62.0)	0.826
No	180	37.7	(32.5–43.1)	158	35.7	(30.4–41.3)	
I don’t know	37	7.7	(5.3–11.2)	35	7.9	(5.3–11.5)	

^a^For this survey question the Fisher’s test P-value was obtained with respect to the “unselected” replies for each vaccine (not shown)

The 95% confidence intervals (CI) are shown

Respondents under both non-intervention and intervention groups had similar awareness about which vaccines pharmacists were authorized to provide to the public (Table 4). Specifically, while a majority were aware of pharmacists’ authority to provide influenza vaccine (68.4% and 68.8% respectively), there was a decreasing awareness for travel vaccines (33.2% and 32.7%), shingles (23.6% and 26.7%), pertussis (12.7% and 11.9%), and meningococcal ACWY/C (9.6% and 8.3%) and B (9.2% and 8.1%) (all had P = NSS between non-intervention and intervention groups). Pooled results from both intervention/non-intervention groups are shown in Fig. S1. In sum, outside of influenza, there was a lack of awareness about the types of vaccines provided by pharmacists, and this did not differ between non-intervention and intervention groups.

Furthermore, 37.7% and 35.5% of respondents from the non-intervention and intervention groups respectively (P = NSS, Table 4) indicated that they had not been informed by any healthcare provider (family physician, nurse, or pharmacist) about the types of vaccines they should receive. In turn, 39.7% and 35.4% agreed that they would be more comfortable receiving a vaccine from a pharmacist if recommended by a physician, while 33.0% and 31.7% were more comfortable if recommended by a nurse (P = NSS in both contrasts, Table 4).

### Willingness to pay for vaccines

**Fig. 3:**
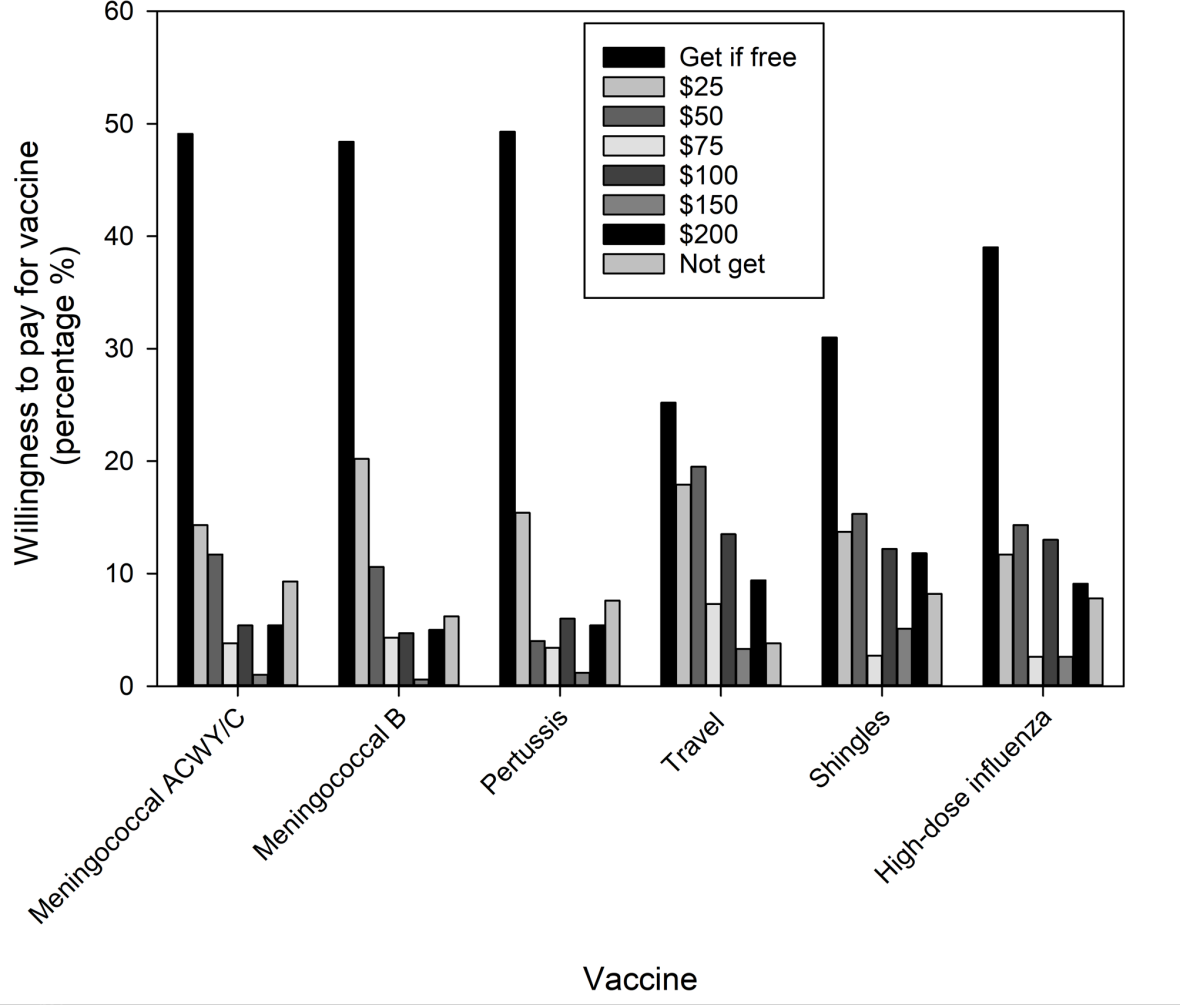
Percent of respondents willing to pay for a vaccine (and cost) or receive if free. Respondents were directed to provide only one response among the options. The percent of respondents not willing to receive a vaccine is also shown

The price that respondents were willing to pay, in a range from free up to $200, per vaccine was similar between the non-intervention and intervention groups (*P* ≥ 0.231 for all vaccines under study). The willingness to pay for each vaccine for all respondents is shown in Fig. 3, excluding influenza vaccine whose cost has historically been covered by Public Health in Canada, including at the onset of this study [44, 45]. This includes the percentage of all respondents willing to pay costs ranging between $25 to $200, those who would only take the vaccine if free, and those who would not receive the vaccine. The percentages of respondents willing to pay for each vaccine, pooled across all cost points, are shown in Fig. S2. We found that the vaccines most likely to be obtained if provided for free included pertussis (49.3%), and meningococcal ACWY/C (49.1%) and B (48.4%; Fig. 3, Fig. S2). When considering all pooled cost points, respondents were most willing to pay for the travel vaccines (70.9%) followed by shingles (60.8%) and high-dose influenza (53.3%) (Fig. S2). Each vaccine had a relatively small subset of respondents (3.8% to 9.3%, depending on vaccine) that would not receive the vaccine regardless of cost, or even if free (Fig. 3, Fig. S2). For each studied vaccine, 90.7% or more of the respondents would receive the vaccine, when pooling those that would take it for free or at a cost (Fig. S2). From combining this result with the percentage of respondents that had received each vaccine in Fig. 1 (Y-axis), there was a marked gap in the percentage of people willing to take each vaccine (≥ 90.7%) versus those that actually have received the vaccine, the latter of which was below 50% for some vaccines (pertussis, shingles, high-dose influenza, and meningococcal B and ACWY/C, Fig. 1).

### Sources of vaccination information

**Fig. 4:**
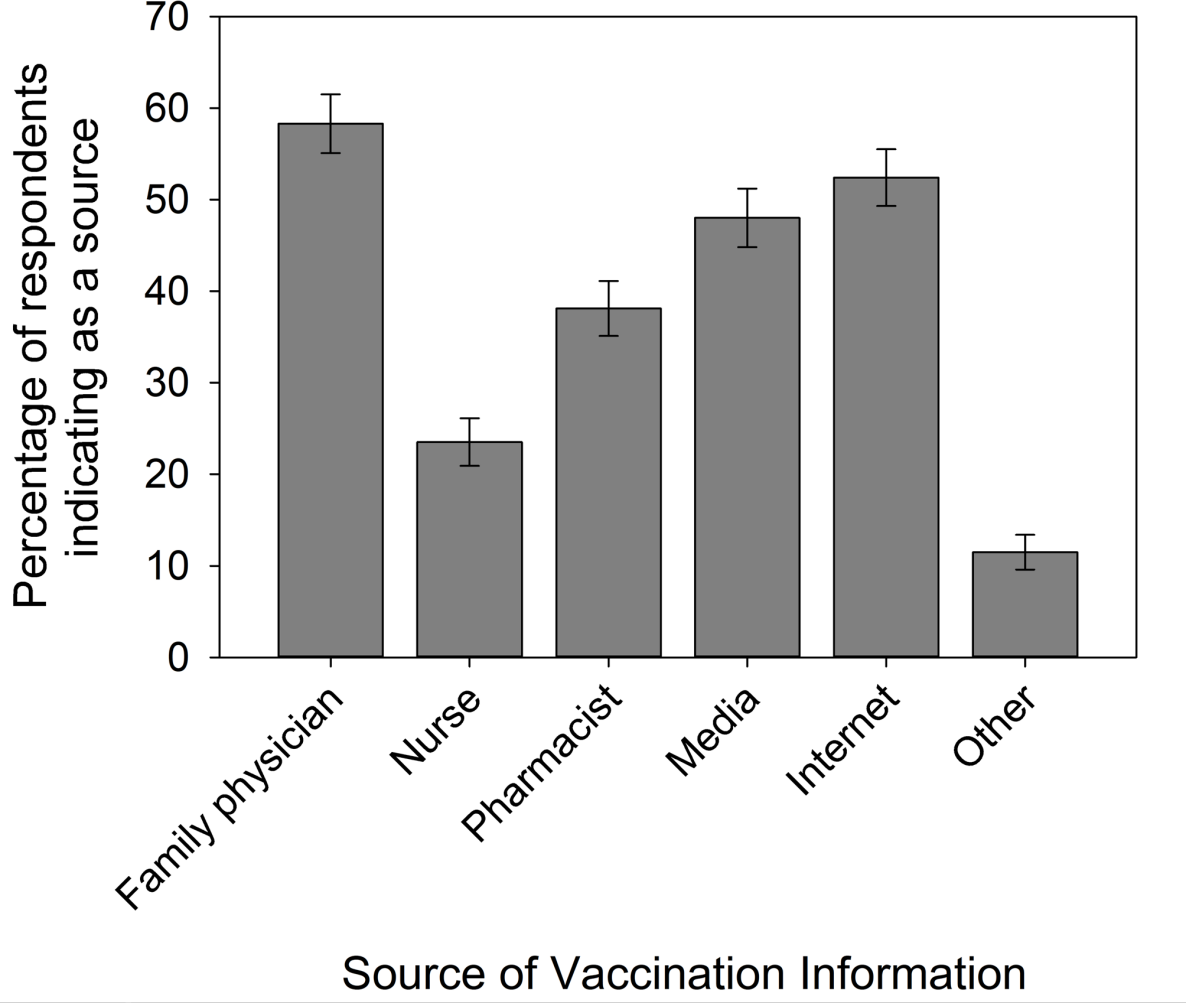
The sources of vaccine information used by respondents (percent per source). *n* = 992. Bars = 95% confidence intervals

We examined where respondents obtained their vaccine information to identify ways to improve the use of pharmacists as immunizers. Respondents indicated that their primary source(s) of information was from their family physician (58.3%), followed closely by the internet (52.4%) and media (48.0%), while pharmacists (38.1%) and nurses (23.5%) were relatively less common sources (Fig. 4). There were some trends when comparing the intervention and non-intervention groups: for instance, there was a decrease of 6.2 PP (or 12.2% relative to the non-intervention value) in the use of media in the intervention group (Fisher’s test, *P* = 0.056), and an increase in the use of physicians by 3.8 PP (6.7%), and of nurses by 4.5 PP (21.1%, however P = NSS in both cases, Table S3). Nonetheless nurses remained the least used information source for the intervention and non-intervention groups, while the use of pharmacists and the internet were each highly similar between groups (Table S3).

Using multivariate analysis, we assessed each source of information (physicians, nurses, pharmacists, media, internet) to ascertain the characteristics of respondents that favored each source (across all respondents; factors with *P* < 0.05 in Table 5; extended results in Table S4). We found that respondents that did not have trust in the scientific knowledge on vaccines had five times higher odds of using the internet as a source of vaccination information than those that had no opinion (odds ratios are in Table 5). Respondents that did not have a family physician (13.9% of all respondents) had two- and three-times higher odds of using the media and internet as a source of vaccine information, respectively, than those that did have a physician (Table 5). Further, individuals that had a physician had two-fold higher odds of using a physician for vaccine information than those that did not (Table 5). Respondents who visited the pharmacy more than once per month (or > 12 times per year) and 7–12 times per year had more than four- and two-times higher odds respectively of receiving their vaccination information from pharmacists, than those who visited less than once a year (Table 5). Thus, pharmacists provide a significant source of vaccine information for returning patients (Table 5).

**Table 5:** Factors linked to information sources using multiple regression (*P* < 0.05), with odds ratios per response category

Information Source (and survey query)	*P*-value	Response	Odds ratio (and 95% CI)
**Family Physician**			
Have a family physician	0.008	No	1
		Yes	2.08 (1.28,3.40)
**Pharmacist**			
Frequency of pharmacy visits	0.016	< once a year	1
		2–6 times a year	1.85 (1.02,3.35)
		7–12 times a year	2.04 (1.07,3.87)
		>once a month	4.50 (1.73,11.68)
**Nurse**			
Age	0.033	18–24 years	1
		25–34 years	0.58 (0.29,1.18)
		35–44 years	0.55 (0.25,1.22)
		45–49 years	0.68 (0.28,1.63)
		50–54 years	0.47 (0.20,1.15)
		55–64 years	0.39 (0.20,0.75)
		≥ 65 years	0.29 (0.13,0.69)
**Media**			
Have a family physician	0.017	No	1
		Yes	0.46 (0.24,0.87)
I trust vaccine recommendations made by public health officials in Canada	0.041	Disagree	1
		Neither	0.91 (0.33,2.51)
		Agree	1.98 (0.99,3.94)
**Internet**			
Have a family physician	0.002	No	1
		Yes	0.33 (0.16,0.66)
I trust current scientific knowledge about vaccines	0.012	Disagree	1
		Neither	0.19 (0.06,0.61)
		Agree	0.57 (0.23,1.42)

Notes: Extended results are provided in Table S4

## Discussion

### Pharmacy interventions and vaccination receipt

The central factor under investigation in most prior pharmacy vaccine intervention studies is the shift in patients’ vaccine receipt, or vaccine behaviors, associated with the interventions [3, 24–29, 46]. Our study used a different approach, and assessed self-reported vaccine receipt among the general public in communities with and without two-years of pharmacy interventions. Although few comparable studies are available to date, that have assessed interventions across multiple pharmacies on the general public, a two-year intervention was conducted for seasonal influenza vaccine in rural regions of British Columbia, which targeted seniors and other at-risk groups. In that study, pharmacy-based influenza clinics were held and were promoted by invitations sent to pharmacy patients, posters, and by media events (no activities in the control communities); the intervention group was linked to increased community-wide influenza vaccine uptake in people 65 years of age and older, but no change was observed among at-risk individuals aged 2 to 64 [47]. Herein, we showed that the pharmacy intervention group was statistically significantly associated with individuals’ responses about their self-reported vaccination status for three vaccines, with an increased awareness of their vaccination status and an increase in uptake for meningococcal ACWY/C and travel vaccines, and a decline in the use of the shingles vaccine (Table 3). Together, the prior assessment [47], and the present study, each demonstrate that community-wide pharmacy interventions may influence vaccine behaviors by the public (in that case for influenza vaccine uptake in seniors [47]).

The shifts in vaccination behaviors observed here are consistent with trends reported from more narrowly focused and small-scale pharmacist interventions that have usually studied uptake of one (or two) vaccines of targeted sets of known patients/individuals before and after pharmacy intervention or relative to a control group [3, 24–29, 46]. Such studies have typically shown that interventions are linked to improvements in uptake; for instance, for influenza, an increase in uptake has ranged from a small 1.03-fold fold increase (albeit non-significant [24]) to more than a three-fold improvement in vaccine receipt [28]) (see similar studies, including other vaccines in [3, 25–27, 29, 46]). While this type of research has provided significant insights on pharmacy interventions and vaccine uptake in targeted sets of individuals, our investigation is different in that we used a broader approach and showed that vaccine knowledge and behaviors (including uptake) of the general public were associated with the intervention status (non-intervention versus intervention) of their communities.

### Findings from combined non-intervention and intervention surveys

Several strategies are suggested by our findings from the combined intervention and non-intervention analyses that may facilitate the public’s use of pharmacists as immunizers, as described below.

### Proactive offering of vaccines

The findings that those vaccines more commonly offered within the communities tended to have greater uptake among respondents (Fig. 1, Table S1) suggests that pharmacists should proactively offer suitable vaccines to their patients (registered and walk-ins). This notion is in alignment with a recent metanalysis of qualitative studies on pharmacy interventions, which suggested that proactive outreach by pharmacists should include verbal communication, check-ins with patients on their vaccine status, providing recommendations, and promotion of vaccination days [24]. Further, the proactive offering of COVID-19 vaccines to the public during the pandemic is thought to have contributed to the widespread uptake of those vaccines [48]. In the present study, pharmacists were permitted to use their professional discretion with respect to which interventions were employed in their pharmacy for their patients. However, these findings (Fig. 1) demonstrate that routine and proactive offering of vaccines to patients should be a targeted and primary strategy used by pharmacists to improve vaccine uptake.

### Counselling on public health recommendations

The findings that an awareness of NACI recommendations was strongly correlated to respondents’ intention to receive a particular vaccine in the future (Fig. 2), suggests that educating the public about Public Health recommendations [23, 33], including counselling by pharmacists (as well as emails, posters), may facilitate greater vaccine uptake. Further, highlighting the rigor used by NACI in development of their recommendations should be emphasized, such as describing how NACI makes their decisions based on rigorous literature reviews on the effectiveness/efficacy of each vaccine written by expert scientists in immunology [49, 50], that may address any patient concerns about a particular vaccine [48]. Thus, both proactive offering of vaccines, combined with counselling on NACI recommendations, provide promising avenues to improve vaccine uptake from pharmacists.

### Increased awareness of vaccines administered by pharmacists

Only a minority of respondents studied herein were aware that pharmacists could provide vaccines other than influenza vaccine (Fig. S1). Remarkably, less than 15% of all study respondents were aware that pharmacists can administer meningococcal ACWY/C and B and pertussis vaccines (Fig. S1). Importantly, family physicians, nurses, and pharmacists, each comprise substantial sources of information about vaccination for the public (Fig. 4) and some respondents were more apt to receive a vaccine by a pharmacist when recommended by a physician/nurse (Table 4). Thus, support of other health professionals is integral to expanding the use of pharmacists as immunizers. Our prior research showed that while many physicians and nurses support pharmacists’ roles in providing vaccines to adults in these same communities in NS and NB, this was not universal, and 61.7% and 72.2% support pharmacists as immunizers of adults, respectively [51] (see also [52]). Thus, counselling of the public by physicians and nurses on the types of vaccines provided by pharmacists may help improve vaccination rates. Pharmacists themselves may also expand the public’s awareness of the types of vaccines they provide, particularly via communications with their repeat patients, who indicated that pharmacists were a favored source of vaccination information (Table 5).

### Public Health coverage of vaccine costs

Vaccine cost was a barrier to vaccine uptake in both the non-intervention and intervention groups (Fig. 3). In Canada, meningococcal ACWY/C and Tdap are publicly funded under the childhood vaccine schedules. The Tdap vaccine is recommended in adulthood (including pregnancy) and has recently expanded (beyond when provided by physicians/nurses) to become available at no personal cost in NS when administered by a pharmacist for individuals with a provincial health card (but only covered in NB when provided by a physician/nurse [53–55]). Given that people are likely motivated by their travel plans, it may not be surprising that a majority of respondents were willing pay ($25 to $200) for travel vaccines (70.9%, Fig. S2). While adult influenza vaccine is publicly funded in Canada, this coverage has recently extended to include high-dose influenza vaccines for those 65 years and older in NS (since 2023) and NB (since 2021), including when provided by pharmacists [44, 45]. For meningococcal B, shortly after our surveys were completed (2021), this vaccine became publicly funded in NS, including when received from pharmacists, for those under 25 years of age deemed at risk, such as those in congregate living at university dorms and military barracks [56]. While ≤ 36.7% of respondents in this age group in our study had actually received the meningococcal B vaccine (intervention and non-intervention groups, Table 3), our data suggest that 93.8% of all respondents in this age group would be willing to receive the vaccine (after pooling free and paid, Fig. S2), and thus the recent public funding has the potential to close this vaccination gap.

The shingles vaccine, recommended for people aged 50 years and above by NACI [23, 33], is not publicly funded in NB, and has very recently become funded in NS (May 2025) for residents aged 65 years or older [57]. For members of the public without public or private coverage of costs, the shingles vaccine is available at about $175 (CAD) per dose at pharmacies in a two-dose series ($350 total plus injection/prescription fees [58]), and may/may not be covered by individual prescription plans. From our findings in Fig. 3, this level of cost may limit the uptake of the shingles vaccine. Specifically, we found some degree of hesitancy, as the intention to receive the shingles vaccine was low relative to knowledge about the NACI recommendations (Fig. 2), and even though it was a known health threat (Table S2). Shingles vaccine hesitancy has also been ascribed to beliefs that one has personal control of the disease, and concerns about vaccine profiteering [59, 60]. The reduced uptake of shingles vaccine in the intervention group examined here (Table 3) may suggest that barriers such as high out-of-pocket costs, limited public funding, or persistent misconceptions about shingles vaccination, were not overcome by the interventions in the studied communities, and potentially hesitancy was exacerbated. Fortunately, our results suggest that the proactive offering of the shingles vaccine by pharmacists (Fig. 1) may be an effective strategy for overcoming those barriers. In terms of costs, the recent public funding in NS to provide the shingles vaccine for seniors (≥ 65 years; that occurred after the surveys here), including when provided by pharmacists [57], largely removes the cost barrier for vaccine uptake for this subset the public. Taken together, the recent governmental focus on supporting public funding for some of the studied vaccines here (meningococcal B, standard and high-dose influenza, adult Tdap, and shingles), including covering costs when administered by pharmacists, is apt to improve uptake.

### Reaching the underserved population and vaccine skeptics

Improvement of the use of pharmacists as immunizers may also require strategies towards those members of the public that are underserved by the healthcare systems. For instance, we found that a subset of respondents (13.9%) did not have access to a family physician (concurring with other estimates [11]), which disproportionately includes young adults, those within the lowest income group [12], and those residing in rural and remote communities [61]. This presents a vaccine information challenge, given that physicians were at least two-fold more commonly used as an information source for those with access (than those without access, Table 5). For these underserved individuals, outreach strategies may involve the use of targeted media and internet approaches; for example, advertisements indicating that pharmacists can provide the vaccine against meningococcus B - a disease that disproportionally affects those under age 25 [12], a group that is least likely to have a physician [11].

In addition to underserved individuals, those respondents most skeptical of vaccination (i.e., those that did not trust scientific knowledge of vaccines, Table 5) tended to use the internet as their vaccine information source. Growing evidence suggests that internet sources, including social media and emails, may positively influence perspectives on vaccines. For instance, a physician and pharmacist intervention that used Facebook coaching sessions with vaccine hesitant respondents, reduced vaccine hesitancy for COVID-19 from 64.3% to 20.1% [62]. Moreover, vaccine educational information provided on social media platforms, including YouTube, has been linked to improved knowledge, attitudes, and behaviors of users towards vaccination [63]. Email reminders have also been shown to improve uptake of vaccines, including the annual influenza vaccine [64, 65]. Thus, the targeted use of social media campaigns and emails may provide a meaningful strategy to combat knowledge barriers among vaccine-hesitant individuals, thereby addressing imprecise information online [66].

### Trust in pharmacists

Reassuringly, both the non-intervention and intervention groups shared high regard for pharmacists as immunizers, with the vast majority of respondents expressing an awareness of their training to administer vaccines (>92%) and comfort in receiving vaccines from pharmacists (>85%, Table 4). The public’s trust in pharmacists has been described previously [1, 8, 31] and provides a solid foundation for the expansion of pharmacists’ roles as immunizers [8, 31]. Further, the convenience of pharmacists in providing vaccines, which was indicated by many respondents studied here adds to their attractiveness as immunizers [8]. Thus, the trust in and accessibility of pharmacists are beneficial for employing all strategies described above.

### Remaining challenges with improving the use of pharmacists as immunizers

Proactive outreach to patients by pharmacists has been previously suggested to be essential to improving immunization rates of the “movable middle”, that is, the group of people that have questions but are open to changing their vaccination intentions based on advice of a trusted health professional [48]. Although many pharmacists are willing to proactively engage patients in vaccination, some research suggests that they prefer to be directly compensated for immunization-related tasks (fees not being paid to the pharmacy) for the added responsibilities of reviewing patient histories, patient counselling, and administering vaccines [67]. In addition, some pharmacists have concerns about the challenges and time involved in interacting with vaccine-hesitant individuals [48, 67, 68], and managing vaccine adverse events and paperwork/billing [8]. Addressing these outstanding challenges should be a part of a strategy to enhance the role of pharmacists as immunizers.

A significant barrier to immunization of the public by pharmacists is geographic location. While residents of the communities studied herein had access to their local pharmacies, there remains a substantial logistical challenge for people living in rural and isolated communities, whereby physician access is limited (including poor availability for in-person visits, by phone, video conference, or by physician travel [69]), and pharmacies are not always in close proximity. For instance, in NS, although nearly all residents in urban settings have access to a pharmacy, only 53.3% of rural residents are within 5 km of a pharmacy [70]. Thus, in rural regions, individuals without personal or public transportation access have limited/few options for vaccination, even by pharmacists [71]. One potential approach that may improve vaccination access in remote regions may be through publicly funded programs to allow pharmacists to travel to patients for assessments and for vaccinations, such as mobile clinics [72, 73].

### Study limitations

There are several limitations to the present study. The demographics of respondents (Table 2) were skewed towards younger individuals (Table 2), that may have influenced some findings. However, our survey included age-controlled queries for meningococcal B, shingles and high-dose influenza vaccines, aimed at mitigating any such effects. The respondents also disproportionately included females (Table 2), whose experiences may differ from males and gender-diverse individuals. Females may also be more apt to have a primary-care physician [12], which may affect one’s vaccine information sources and experiences (Table 5). Further, it is conceivable that people who volunteered to complete the surveys were more vaccine positive than those who chose not to participate. In addition, the COVID-19 pandemic occurred between the interventions (2017 to 2019) and the survey period, and thus individual responses may have been influenced by the growing roles of pharmacists in COVID-19 vaccination.

While we included a range of intervention methods (vaccine of the week, linking vaccine information to other services, posters), our data suggest that future interventions should target a narrower range of strategies. For instance, these should involve outreach to individuals without a family physician, and especially for pharmacists to proactivity offer suitable vaccines (i.e., influenza vaccine before/during influenza season, age-related vaccines to those within the recommended age range, or vaccines recommended for immunocompromised individuals) and provide counsel on Public Health recommendations, including for repeat visitors to the pharmacy who tend to rely on vaccine information from their pharmacists (Table 5). Another limitation is that surveys, as used here, are innately developed from the researchers’ viewpoint, such that respondents are limited to the responses provided per query, which may not always reflect their full range of experiences. Thus, further community-wide intervention studies using qualitative approaches, with participant-led data (for instance, open-ended questions for participants to respond in their own words (cf. [52]), may reveal a wider breadth of participants’ perspectives than reported here. Finally, it should be noted that while we found that five studied vaccines showed improvements in uptake in the intervention as compared to the non-intervention group (influenza, meningococcal ACWY/C and B, travel, and pertussis increased in uptake, but not shingles; high-dose influenza was excluded due to low n values, Table 3), we did not make any conclusions on this pattern, as not all of the contrasts were statistically significant (Table 3). Larger n values in further studies may help decipher these relationships.

### Benefits of pharmacists as immunizers for public health

Expanding the use of pharmacists as immunizers by the public provides a pathway to improve vaccine coverage for vaccine-preventable diseases [18, 19, 21], and in turn, to reduce morbidity and mortality within communities and to lower the burden of disease to workforces and healthcare services [1, 5, 6, 74]. For example, enhanced uptake of seasonal influenza vaccines from pharmacists may help to reach the Canadian Public Health goal of 80% coverage of adults [7], and substantially reduce the spread of the virus, including to those people most vulnerable to complications (e.g., pneumonia, hypoxia, myocarditis), such as the elderly, obese, and with immune conditions [75]. Heightened influenza vaccination rates are apt to reduce worker absenteeism, including within the healthcare sector [6, 74], and to diminish the burden of influenza patients to hospitals and family physicians every year, which currently costs the public financially (under publicly funded healthcare), and also contributes to the chronic overcrowding of healthcare facilities [76, 77]. When considering the additional types of vaccines that are provided by pharmacists, such as those studied here (meningococcal ACWY/C and B, travel, and shingles), identifying strategies to improve the use of pharmacists as immunizers is essential to improved public health, and for reduced economic burdens of disease on communities.

## Conclusions

Taken together, the present results showed that pharmacy interventions applied over two years and across multiple pharmacies within communities in NS and NB were associated with individuals’ awareness of their vaccination status and vaccine uptake (Table 3). The combined results from intervention and non-intervention groups revealed significant strategies to help improve the use of pharmacists as immunizers. These include pharmacists proactively offering suitable vaccines to patients (Fig. 1, Table S1), informing patients of NACI recommendations (Fig. 2), and widening the public’s awareness of the vaccines that pharmacists are authorized to administer through discussions with patients (that also includes advocacy from physicians and nurses, Fig. 4; Tables 4 and 5). In addition, expanding pharmacists’ roles as immunizers may be facilitated by public funding of NACI recommended vaccines (Fig. 3), and by provision of media and internet vaccine campaigns aimed to reach those individuals without a family physician and those most skeptical of vaccination (Table 5). Fortunately, the positive attitudes and widespread trust of the public towards pharmacists in the studied communities (Table 4), represent major advantages in achieving the goal of expanding their roles as immunizers. The strategies identified herein are relevant not only to Canada, but also to the growing number of countries where pharmacists serve as immunizers.

## Supplementary Information

Supplementary material 1.

## Data Availability

The datasets may be obtained from the corresponding author upon reasonable request.

## Abbreviations

CAD: Canadian dollar
CCfV: Canadian Center for Vaccinology
KABB: knowledge, attitudes, beliefs, and behaviors
Meningococcal ACWY/C vaccine: Meningococcal vaccine associated with serogroups A, C, W, or Y/Meningococcal C vaccine
Meningococcal B vaccine: Meningococcal vaccine associated with serogroup B
NACI: National Advisory Committee on Immunization
NB: New Brunswick
NS: Nova Scotia
PP: percentage points
Tdap vaccine: tetanus, diphtheria, and acellular pertussis vaccine
