# Supplementary material for: The impact of two-year community-wide pharmacy interventions on the public’s knowledge, attitudes, beliefs and behaviors about pharmacists as immunizers
Source: BMC Public Health. 2025 Dec 5;25:4221. doi: 10.1186/s12889-025-24906-3 (PMC12681092; doi:10.1186/s12889-025-24906-3)
Supplement: Supplementary file 1 — Supplementary material 1. [file 12889_2025_24906_MOESM1_ESM.docx]

**ADDITIONAL FILE 1**

**for**

**The impact of two-year community-wide pharmacy interventions on the public’s knowledge, attitudes, beliefs and behaviors about pharmacists**

**as immunizers**

**Authors**: DM Halperin, AM Di Castri, JE Isenor, WK Lowe, L Ye, MS Kervin, CA Whittle, SA Halperin

**
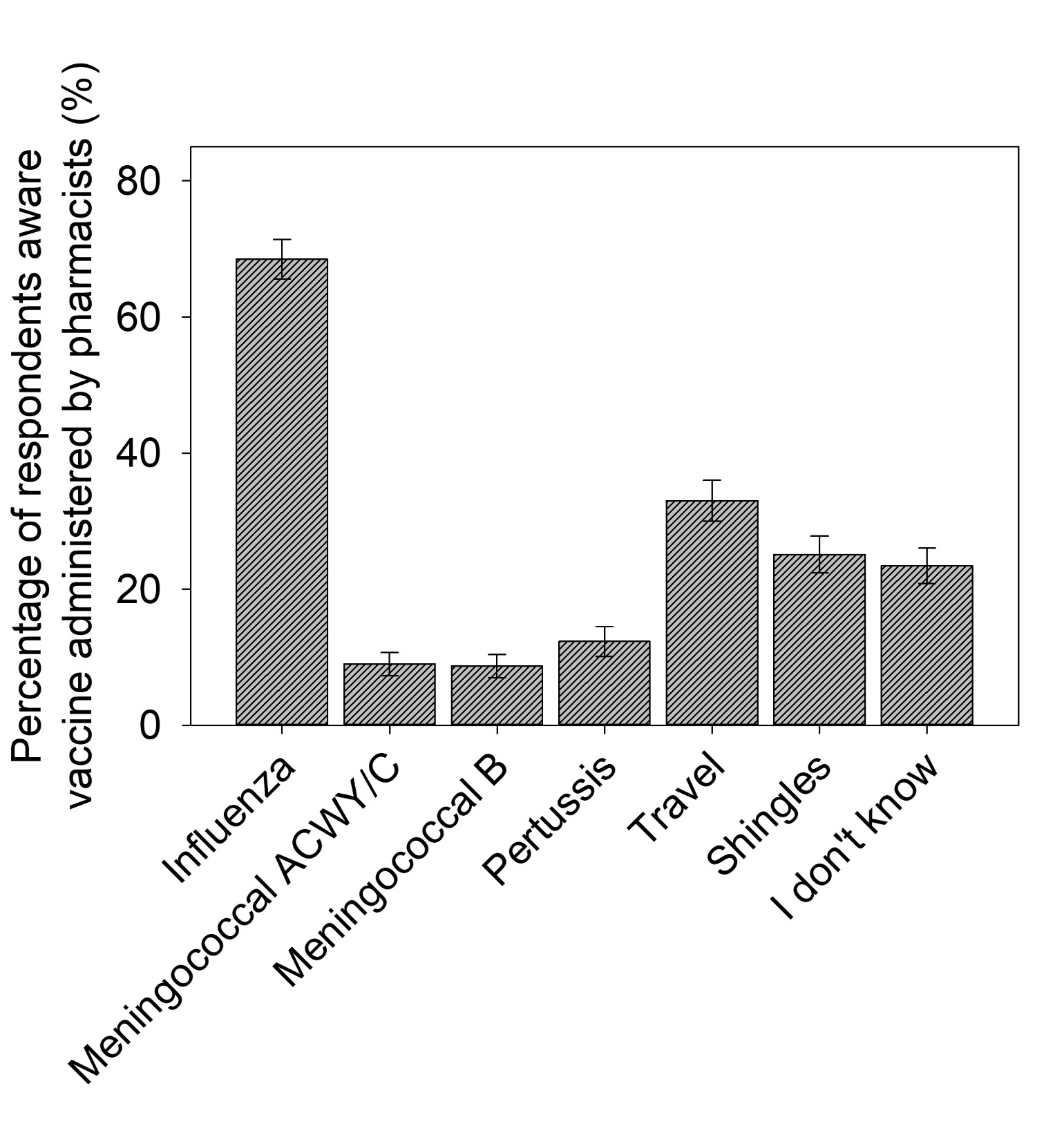
**

**Figure S1.** The percent of respondents that were aware that pharmacists have the authority to provide each vaccine. Note that high-dose influenza was not included as a separate answer from influenza for this question in the survey. Bars represent the 95% confidence intervals.


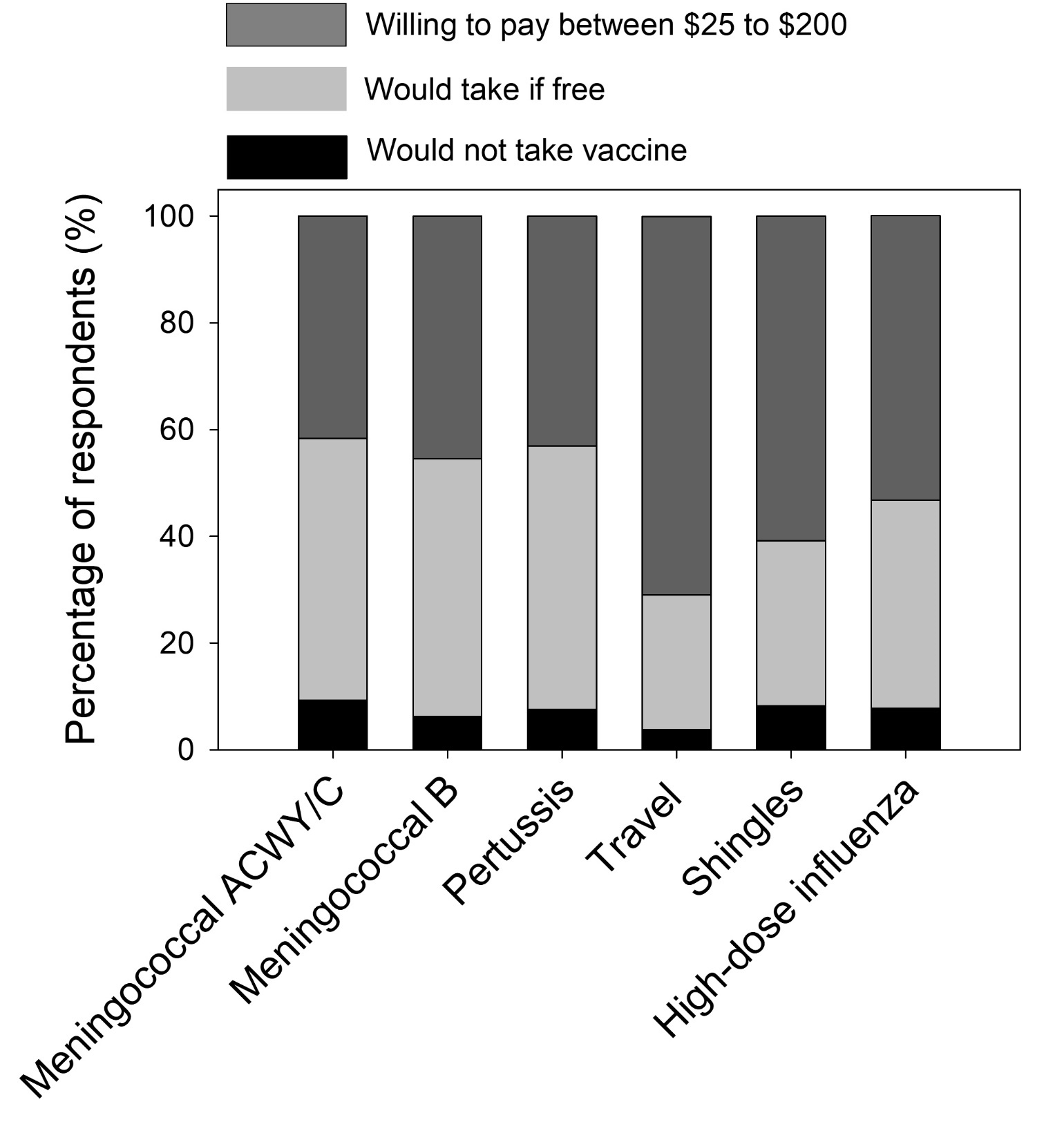


**Figure S2.** The percentage of respondents that would not take the vaccine, would take if free and would be willing to pay for a vaccine in 2021 surveys.

**Table S1.** Factors linked to receipt of a vaccine using multiple regression (P<0.05) and including the odds ratios per response category. The ratios described in the main text are in bold.

| **Vaccine received (and survey query)** | **P-value** | **Response** | **Odds ratio and 95% CI)** |
| --- | --- | --- | --- |
|  |  |  |  |
| **Influenza** |  |  |  |
| **Offered an influenza vaccine** | **0.009** | **No** | **1** |
|  |  | **Yes** | **9.93 (2.29, 43.05)** |
|  |  | **I don't know** | **4.68 (0.27, 81.20)** |
| Ever offered a meningococcal (meningitis) ACWY/C vaccine | 0.009 | No (Ref) | 1 |
|  |  | Yes | 0.35 (0.16,0.77) |
|  |  | I don't know | 1.18 (0.38,3.64) |
| Ever offered a Tdap vaccine | 0.009 | No (Ref) | 1 |
|  |  | Yes | 1.96 (0.86,4.48) |
|  |  | I don't know | 0.50 (0.17,1.52) |
| Ever received a vaccination by a pharmacist in a pharmacy | <0.001 | No (Ref) | 1 |
|  |  | Yes | 10.04 (5.48,18.39) |
|  |  | I don't know | 34.98 (2.06, 594.41) |
| Vaccination is important to me | 0.019 | Disagree (Ref) | 1 |
|  |  | Neither | 10.10 (0.67,153.40) |
|  |  | Agree | 22.39 (2.23,224.49) |
| I don't have time to get vaccinated | 0.001 | Disagree (Ref) | 1 |
|  |  | Neither | 0.90 (0.29,2.77) |
|  |  | Agree | 0.11 (0.04,0.36) |
| I am at significant risk for developing influenza | 0.01 | Disagree (Ref) | 1 |
|  |  | Neither | 0.63 (0.32,1.24) |
|  |  | Agree | 2.33 (1.08,5.05) |
| Influenza is rare enough that I no longer need to be vaccinated against it | <0.001 | Disagree (Ref) | 1 |
|  |  | Neither | 0.19 (0.08,0.44) |
|  |  | Agree | 0.86 (0.25,2.94) |
| Obtain information about vaccination from family physician | 0.011 | Unchecked (Ref) | 1 |
|  |  | Yes | 2.27 (1.21,4.29) |
|  |  |  |  |
| **Meningococcal (meningitis) ACWY/C** |  |  |  |
| Age | 0.001 | 18-24 years (Ref) | 1 |
|  |  | 25-34 years of age | 2.84 (0.68,11.88) |
|  |  | 35-44 years of age | 0.37 (0.09,1.57) |
|  |  | 45-49 years of age | 3.45 (0.75,15.90) |
|  |  | 50-54 years of age | 0.09 (0.02,0.43) |
|  |  | 55-64 years of age | 0.30 (0.07,1.34) |
|  |  | ≥65 years | 0.10 (0.01,0.71) |
| **Ever offered a meningococcal (meningitis) ACWY/C vaccine** | **<0.001** | **No (Ref)** | **1** |
|  |  | **Yes** | **859.45 (233,3167)** |
|  |  | **I don't know** | **12.16 (3.58,41.32)** |
|  |  |  |  |
| **Tdap** |  |  |  |
| Age | <0.001 | 18-24 years (Ref) | 1 |
|  |  | 25-34 years of age | 10.70 (3.55,32.29) |
|  |  | 35-44 years of age | 18.30 (5.74,58.31) |
|  |  | 45-49 years of age | 18.77 (5.29,66.66) |
|  |  | 50-54 years of age | 11.86 (3.73,37.64) |
|  |  | 55-64 years of age | 22.60 (7.93,64.44) |
|  |  | ≥65 years | 10.05 (3.79,26.66) |
| **Ever offered a Tdap vaccine** | **<0.001** | **No (Ref)** | **1** |
|  |  | **Yes** | **17.99 (8.36,38.70)** |
|  |  | I don't know | 1.38 (0.51,3.73) |
| Meningitis (meningococcal B) is a vaccine currently administered by pharmacists | 0.009 | Unchecked (Ref) | 1 |
|  |  | Checked | 0.41 (0.21,0.80) |
| Have any of your healthcare providers (e.g. family physician, nurse, pharmacist) informed you of vaccines you should receive? | <0.001 | No (Ref) | 1 |
|  |  | Yes | 4.17 (2.25,7.72) |
|  |  | I don't know | 1.04 (0.34,3.18) |
| Receiving vaccines protects the people I care about from getting ill | <0.001 | Disagree (Ref) | 1 |
|  | 0.028 | Neither | 10.96 (1.44,83.39) |
|  |  | Agree | 8.19 (1.69,39.57) |
| **Aware that NACI recommends adults receive a vaccine against pertussis** | **<0.001** | **No (Ref)** | **1** |
|  |  | **yes** | **6.60 (3.32,13.14)** |
| Know which vaccines to receive based on public health recommendations | 0.019 | No (Ref) | 1 |
|  |  | yes | 0.48 (0.26,0.89) |

**Table S2.** The percent of respondents that agree and disagreed that each disease was a health threat. The 95% confidence interval (CI) is shown.

| **Perception of Illness as a Threat** | **N** | **Percent**  **Agree** | **CI** | **Percent**  **Disagree** | **CI** |
| --- | --- | --- | --- | --- | --- |
|  |  |  |  |  |  |
| Influenza poses a serious threat to the health of adults | 873 | 78.7 | (75.2,81.8) | 8.9 | (6.9,11.5) |
| Meningococcal meningitis poses a serious threat to the health of adults | 873 | 55.7 | (51.2,59.2) | 5.3 | (3.7,7.4) |
| Whooping cough (pertussis) poses a serious threat to the health of adults | 873 | 45.6 | (41.6,49.6) | 14.8 | (12.1,17.9) |
| Travel: Hepatitis A poses a serious threat to the health of travelers to high-risk areas | 845 | 82.4 | (79,85.3) | 1.5 | (0.8,2.9) |
| Travel: Hepatitis B poses a serious threat to the health of travelers to high-risk areas | 845 | 77.3 | (73.7,80.5) | 2.8 | (1.8,4.6) |
| Travel: Typhoid poses a serious threat to the health of travelers to high-risk areas | 844 | 70.5 | (66.6,74.1) | 3.4 | (2.2,5.3) |
| Shingles (herpes zoster) poses a serious threat to the health of adults | 845 | 82.8 | (79.5,85.7) | 3.7 | (2.4,5.6) |
|  |  |  |  |  |  |

Notes: The values for “I don’t know” are not shown but are the remainder of results per question. The number of respondents per question are shown, noting that not all participants (n=992) replied to all queries.

**Table S3.** The respondents’ replies on their sources of information for the non-intervention and intervention groups. The percentage point difference and the percent difference of intervention relative to non-intervention group is shown. A negative value indicates a higher percentage in the non-intervention group.

| **Source of Information** | **Yes/No** | **Non-intervention** | |  | **Intervention** | | **P** |  | **Percent point diff** | **Percent diff (%)** |
| --- | --- | --- | --- | --- | --- | --- | --- | --- | --- | --- |
|  |  | **N** | **Percent** |  | **N** | **Percent** |  |  |  |  |
| Family physician | No | 223 | 43.6 |  | 191 | 39.8 | 0.246 |  |  |  |
|  | **Yes** | **289** | **56.4** |  | **289** | **60.2** |  |  | **3.8** | **6.7** |
|  |  |  |  |  |  |  |  |  |  |  |
| Nurse | No | 403 | 78.7 |  | 356 | 74.2 | 0.099 |  |  |  |
|  | **Yes** | **109** | **21.3** |  | **124** | **25.8** |  |  | **4.5** | **21.1** |
|  |  |  |  |  |  |  |  |  |  |  |
| Pharmacist | No | 320 | 62.5 |  | 294 | 61.3 | 0.695 |  |  |  |
|  | **Yes** | **192** | **37.5** |  | **186** | **38.8** |  |  | **1.3** | **3.5** |
|  |  |  |  |  |  |  |  |  |  |  |
| Media | No | 251 | 49 |  | 265 | 55.2 | 0.056 |  |  |  |
|  | **Yes** | **261** | **51** |  | **215** | **44.8** |  |  | **-6.2** | **-12.2** |
|  |  |  |  |  |  |  |  |  |  |  |
| Internet | No | 243 | 47.5 |  | 229 | 47.7 | 0.949 |  |  |  |
|  | **Yes** | **269** | **52.5** |  | **251** | **52.3** |  |  | **-0.2** | **-0.4** |
|  |  |  |  |  |  |  |  |  |  |  |
| Other | No | 444 | 86.7 |  | 434 | 90.4 | 0.073 |  |  |  |
|  | **Yes** | **68** | **13.3** |  | **46** | **9.6** |  |  | **-3.7** | **-27.8** |

**Table S4.** Factors linked to sources of information using multiple regression (P<0.05) and including the odds ratios per response category. Extended results for Table 5 in the main text.

| **Information Source (and survey query)** | **P-value** | **Response** | **Odds ratio and 95% CI)** |
| --- | --- | --- | --- |
|  |  |  |  |
| **Family Physician** |  |  |  |
| Income | 0.005 | <$39,000 (Ref) | 1 |
|  |  | $39,000-<$70,000 | 0.86 (0.35,2.09) |
|  |  | $70,000-<$90,000 | 0.26 (0.11,0.62) |
|  |  | $90,000-<$125,000 | 0.60 (0.24,1.49) |
|  |  | ≥$125,000 | 0.29 (0.13,0.65) |
| Have a family physician |  | No (Ref) | 1 |
|  | 0.008 | Yes | 2.08 (1.28,3.40) |
| Frequency of physician visits | 0.008 | < once a year (Ref) | 1 |
|  |  | 2-6 times a year | 2.08 (1.28,3.40) |
|  |  | 7-12 times a year | 0.55 (0.18,1.71) |
|  |  | > once a month | >10 (0.00,>10) |
| Vaccines are easily accessible to me | <0.001 | Disagree (Ref) | 1 |
|  |  | Neither | 2.81 (0.98,8.04) |
|  |  | Agree | 5.79 (2.21,15.14) |
| Whooping cough (pertussis) is rare enough that I no longer need to be vaccinated against it | 0.005 | Disagree (Ref) | 1 |
|  |  | Neither | 0.47 (0.28,0.78) |
|  |  | Agree | 1.38 (0.55,3.45) |
| **Nurse** |  |  |  |
| Age | 0.033 | 18-24 years (Ref) | 1 |
|  |  | 25-34 years of age | 0.58 (0.29,1.18) |
|  |  | 35-44 years of age | 0.55 (0.25,1.22) |
|  |  | 45-49 years of age | 0.68 (0.28,1.63) |
|  |  | 50-54 years of age | 0.47 (0.20,1.15) |
|  |  | 55-64 years of age | 0.39 (0.20,0.75) |
|  |  | ≥65 years | 0.29 (0.13,0.69) |
| Whooping cough (pertussis) poses a serious threat to the health of adults | 0.031 | Disagree (Ref) | 1 |
|  |  | Neither | 0.72 (0.36,1.43) |
| Whooping cough (pertussis) poses a serious threat to the health of adults | | Agree | 1.42 (0.75,2.67) |
| I trust vaccine recommendations made by public health officials in Canada | 0.02 | Disagree (Ref) | 1 |
|  |  | Neither | 0.43 (0.08,2.33) |
|  |  | Agree | 2.28 (0.95,5.49) |
| **Pharmacist** |  |  |  |
| Frequency of pharmacy visits | 0.016 | < once a year (Ref) | 1 |
|  |  | 2-6 times a year | 1.85 (1.02,3.35) |
|  |  | 7-12 times a year | 2.04 (1.07,3.87) |
|  |  | > once a month | 4.50 (1.73,11.68) |
| Influenza is rare enough that I no longer need to be vaccinated against it | 0.001 | Disagree (Ref) | 1 |
|  |  | Neither | 0.32 (0.16,0.61) |
|  |  | Agree | 0.44 (0.17,1.15) |
| **Media** |  |  |  |
| Have a family physician |  | No (Ref) | 1 |
|  | 0.017 | Yes | 0.46 (0.24,0.87) |
| Meningococcus (meningitis) is rare enough that I no longer need to be vaccinated against it | 0.009 | Disagree (Ref) | 1 |
|  |  | Neither | 1.87 (1.23,2.87) |
|  |  | Agree | 1.98 (0.86,4.52) |
| I trust vaccine recommendations made by public health officials in Canada | 0.041 | Disagree (Ref) | 1 |
|  |  | Neither | 0.91 (0.33,2.51) |
|  |  | Agree | 1.98 (0.99,3.94) |
| **Internet** |  |  |  |
| Have a family physician |  | No (Ref) | 1 |
|  | 0.002 | Yes | 0.33 (0.16,0.66) |
| I have enough information to decide whether or not to get vaccinated | 0.02 | Disagree (Ref) | 1 |
|  |  | Neither | 4.31 (1.26,14.70) |
|  |  | Agree | 0.93 (0.45,1.90) |
| I trust current scientific knowledge about vaccines | 0.012 | Disagree (Ref) | 1 |
|  |  | Neither | 0.19 (0.06,0.61) |
|  |  | Agree | 0.57 (0.23,1.42) |
